# Clade-specific chromosomal rearrangements and loss of subtelomeric adhesins in *Candida auris*

**DOI:** 10.1093/genetics/iyab029

**Published:** 2021-03-26

**Authors:** José F Muñoz, Rory M Welsh, Terrance Shea, Dhwani Batra, Lalitha Gade, Dakota Howard, Lori A Rowe, Jacques F Meis, Anastasia P Litvintseva, Christina A Cuomo

**Affiliations:** Broad Institute of MIT and Harvard, Cambridge, MA 02142, USA; Mycotic Diseases Branch, U.S. Department of Health and Human Services, Atlanta, GA, USA; Broad Institute of MIT and Harvard, Cambridge, MA 02142, USA; Division of Scientific Resources, Centers for Disease Control and Prevention, U.S. Department of Health and Human Services, Atlanta, GA, USA; Mycotic Diseases Branch, U.S. Department of Health and Human Services, Atlanta, GA, USA; Division of Scientific Resources, Centers for Disease Control and Prevention, U.S. Department of Health and Human Services, Atlanta, GA, USA; Division of Scientific Resources, Centers for Disease Control and Prevention, U.S. Department of Health and Human Services, Atlanta, GA, USA; Department of Medical Microbiology and Infectious Diseases, Canisius Wilhelmina Hospital, Center of Expertise in Mycology Radboudumc/CWZ, Nijmegen, The Netherlands; Mycotic Diseases Branch, U.S. Department of Health and Human Services, Atlanta, GA, USA; Broad Institute of MIT and Harvard, Cambridge, MA 02142, USA

**Keywords:** Candida auris, fungal genome, chromosome rearrangement, karyotype variation, subtelomeric variation, cell wall proteins, selection

## Abstract

*Candida auris* is an emerging fungal pathogen of rising concern due to global spread, the ability to cause healthcare-associated outbreaks, and antifungal resistance. Genomic analyses revealed that early contemporaneously detected cases of *C. auris* were geographically stratified into four major clades. While Clades I, III, and IV are responsible for ongoing outbreaks of invasive and multidrug-resistant infections, Clade II, also termed the East Asian clade, consists primarily of cases of ear infection, is often susceptible to all antifungal drugs, and has not been associated with outbreaks. Here, we generate chromosome-level assemblies of twelve isolates representing the phylogenetic breadth of these four clades and the only isolate described to date from Clade V. This Clade V genome is highly syntenic with those of Clades I, III, and IV, although the sequence is highly divergent from the other clades. Clade II genomes appear highly rearranged, with translocations occurring near GC-poor regions, and large subtelomeric deletions in most chromosomes, resulting in a substantially different karyotype. Rearrangements and deletion lengths vary across Clade II isolates, including two from a single patient, supporting ongoing genome instability. Deleted subtelomeric regions are enriched in Hyr/Iff-like cell-surface proteins, novel candidate cell wall proteins, and an ALS-like adhesin. Cell wall proteins from these families and other drug-related genes show clade-specific signatures of selection in Clades I, III, and IV. Subtelomeric dynamics and the conservation of cell surface proteins in the clades responsible for global outbreaks causing invasive infections suggest an explanation for the different phenotypes observed between clades.

## Introduction

The emerging fungal pathogen *Candida auris* has been reported in over 40 countries to date and has become a leading cause of invasive candidiasis in some hospitals, often in severely ill patients (van Schalkwyk *et al.* 2019). *C. auris* isolates are commonly resistant to one or more antifungal drugs and can survive for long periods both in the clinical environment and as a commensal on skin (Welsh *et al.* 2017). Initially identified in cases of ear infection in Japan and South Korea (Kim *et al.* 2009; Satoh *et al.* 2009), cases of systemic infection were soon after reported in India, South Africa, and Venezuela (Chowdhary *et al.* 2013; Magobo *et al.* 2014; Calvo *et al.* 2016). Initial genomic analysis of the global emergence identified four major genetic groups corresponding to these geographic regions or Clades I, II, III, and IV (Lockhart *et al.* 2017). Subsequent genomic analysis identified a single genetically diverse isolate from Iran representing a potential fifth clade (Chow *et al.* 2019). Clades I, III, and IV are responsible for the ongoing multidrug-resistant and difficult to control outbreaks in healthcare facilities worldwide (Chow *et al.* 2018, 2020). Clade II, also termed the East Asia clade, is predominantly associated with cases of ear infection and appears to be less resistant to antifungals than other clades (Welsh *et al.* 2019), though cases of Clade II infections have been reported on different continents (Chow *et al.* 2020).

Comparisons of global isolates have highlighted clade-specific variation in the levels of drug resistance and the mechanisms that contribute to resistance. While Clade II isolates are typically susceptible to azoles and other major antifungals, nearly all isolates in Clades I and III and roughly half of isolates in Clade IV are resistant to azoles. These resistant isolates contain one of three mutations (Y132F, K143R, and F126L) in the drug target lanosterol 14-α-demethylase (*ERG11*) (Lockhart *et al.* 2017; Chowdhary *et al.* 2018; Chow *et al.* 2020). Clades I and IV isolates also have mutations contributing to fluconazole resistance in the transcriptional factor *TAC1b* that regulates the efflux pump *CDR1* (Rybak *et al.* 2020) and expression of *CDR1* has been shown to be differentially regulated between isolates from Clades III and IV (Mayr *et al.* 2020). An average of seven percent of the isolates from Clades I, III, and IV are resistant to echinocandins and resistance has been associated to a single mutation at S639 (S639Y/F/P) in the hotspot1 of the drug target 1,3-beta-D-glucan synthase (*FKS1*) (Chowdhary *et al.* 2018; Chow *et al.* 2020). Although low susceptibility to amphotericin B is common in Clades I and IV, amphotericin B drug-resistant mutations have not been identified and linked to these clades. Together, this highlights interclade variation in drug resistance mechanisms.

Other than clade-specific drug resistance and correlated mutational profiles, little is known about the mechanisms responsible of the phenotypic differences between clades and the increased ability of *C. auris* to be transmitted and persist in healthcare settings. While genome comparisons with the well-studied species *C. albicans* suggest conservation of mechanisms of biofilm formation and host interaction, in addition to drug resistance, little is known about how variation within *C. auris* contributes to phenotypic differences other than drug resistance. Clade II isolates have primarily been isolated from cases of ear infection whereas Clades I, III and IV isolates are primarily linked to invasive infections (Welsh *et al.* 2019). In addition, Clade II isolates have increased susceptibility to disinfectants (Sexton *et al.* 2020), ultraviolet C light disinfection (Chatterjee *et al.* 2020), are more sensitive to environmental stressors (Heaney *et al.* 2020), and induce a lower cytokine production and have simpler mannans relative to Clades I, III, and IV (Bruno *et al.* 2020). Most of the clade-specific differences described thus far have been based on SNP-based analyses, and this reference-based analysis has not typically analyzed differences in genome structure and stability between *C. auris* isolates. We hypothesized that larger genomic differences could also be linked to phenotypic variation. In other haploid and asexual *Candida* species such as *C. glabrata*, chromosomal rearrangements are associated with antifungal drug resistance and host adaptation (Poláková *et al.* 2009), including differences in the repertoire of cell wall proteins (Carreté *et al.* 2018).

Previous studies have provided evidence of karyotypic variation in *C. auris* and of gene content differences between clades (Muñoz *et al.* 2018; Bravo Ruiz *et al.* 2019). Whole-genome alignment of an assembly of a Clade I isolate with that of a Clade III isolate identified an inversion and two translocations (Muñoz *et al.* 2018), and analysis of chromosomal sizes across a wider set of isolates suggested other major rearrangements between clades (Bravo Ruiz *et al.* 2019). Evidence for gene content variation includes the presence of a gene cluster involved in L-rhamnose utilization only in isolates of Clade III, which correlates with the ability of Clade III isolates to grow on L-rhamnose (Ambaraghassi *et al.* 2019). Here, to better understand the genomic variation between the clades linked to phenotypic differences, we leverage complete reference genomes for isolates from Clades I, II, III, and IV to better sample the diversity of these major clades and for the only isolate reported from Clade V. We find that the genomes of Clade II isolates are highly rearranged and are missing large subtelomeric regions that include candidate cell wall proteins conserved in all of the other clades. Many of these candidate cell wall proteins as well as several genes involved in drug resistance appear under selection in the three global clades causing outbreaks. These findings may help explain the differences in clinical presentation between isolates from this clade and those from the clades causing global outbreaks as well as the striking variation *in vitro* susceptibility to antifungal drugs and to disinfectants that impact patient treatment and infection control.

## Methods

### DNA purification

Twelve *C. auris* strains were selected for long-read sequencing, two from Clade I (B11205, B13916), four from Clade II (B11220, B12043, B11809, and B13463), three from Clade III (B12037, B12631, and B17721) two from Clade IV (B11245 and B12342) and the only isolate described to date from Clade V (B18474). High molecular weight DNA for long-read sequencing was obtained using the Epicentre MasterPure yeast DNA purification kit (MPY80200), and immediately followed by purification using Pacific Biosciences Ampure PB (100-265-900). DNA for Illumina sequencing was extracted using the ZYMO Research ZR Fungal/Bacterial DNA MiniPrep kit.

### Genome sequencing and assembly

For B11220, B12043, B13463, B11245, and B18474 long-read sequencing was generated using an Oxford Nanopore Technology Ligation Sequencing Kit 1 D (SQK-LSK108), sequenced on a MinIon Flow Cell R9.4 or R9.4.1 (FLO-MIN106) and basecalled with Albacore v2.0.2. For B11205, B13916, B11809, B12037, B12631, B17721, and B12342 long reads were generated using Single-molecule real-time (SMRT) sequencing using the PacBio RS II or Sequel SMRT DNA sequencing system (Pacific Biosciences, Menlo Park, CA, USA). The total read depth ranged from 35.2X to 181X. Specific details of long-read sequencing are given in Supplementary Table S1.

Reads were assembled using Canu v1.5 and v1.6 (genomeSize = 12000000; stopOnReadQuality=false; correctedErrorRate = 0.075) and Flye v 2.4.2 (genome-size = 12000000) (Koren *et al.* 2017; Kolmogorov *et al.* 2019; Supplementary Table S1). The most contiguous assembly was obtained with Canu for B11245, B11205, B13916, B12043, B11809, B13463, B12037, B17721, B12342, B18474, and with Flye for B11220 and B12631. A tandem motif (AGACACCACCTA{1,2}GAAA{1,2}CC{1,2}) was identified at contig ends; contig ends missing this motif were aligned to the unassembled contigs and manually extended. The genomes of B12043, B13463, and B18474 underwent initial polishing using Nanopore reads with Medaka version 0.8.1 using medaka_consensus with parameter -m r941_min_high (https://github.com/nanoporetech/medaka). All twelve assemblies had four or five iterations of Illumina read error correction using Pilon v1.23 (Walker *et al.* 2014). Assemblies were aligned to each other and to B8441 and B11221 (Muñoz *et al.* 2018) using NUCmer (MUMmer v3.22) (Kurtz *et al.* 2004), and rearrangement sites were visually inspected for support based on alignments of both long and short reads using Integrative Genomics Viewer (IGV) v2.3.72 (Robinson *et al.* 2011).

### Genome annotation

Gene annotation was performed with BRAKER1 (Hoff *et al.* 2016) using RNA-Seq to improve gene structure predictions, as done for previously reported assemblies (Muñoz *et al.* 2018). Briefly, we mapped RNA-Seq reads to the genome assembly using Tophat2 v. 2.1.1 (Kim *et al.* 2013), and used the alignments to predict genes using BRAKER1 (Hoff *et al.* 2016), which combines GeneMark-ET (Lomsadze *et al.* 2014) and AUGUSTUS (Stanke *et al.* 2008), incorporating RNA-Seq data into unsupervised training and subsequently generates *ab initio* gene predictions. tRNAs were predicted using tRNAscan (Lowe and Eddy 1997) and rRNAs predicted using RNAmmer (Lagesen *et al.* 2007). Genes containing PFAM domains found in repetitive elements or overlapping tRNA/rRNA features were removed. Genes were named and numbered sequentially. For the protein-coding gene name assignment, we combined names from HMMER PFAM/TIGRFAM, Swissprot and Kegg products. For comparative analysis genes were functionally annotated by assigning PFAM domains, GO terms, and KEGG classification using KoalaBlast. HMMER3 (Eddy 2011) was used to identify PFAM domains using release 27. Orthologs were assigned using bidirectional blast and OrthoMCL v1.4 (Markov index 1.5; maximum *e*-value 1e-5) (Li *et al.* 2003). The predicted gene number was highly similar across all *C. auris* genomes, totaling 5,328 for B11220, 5,506 for B11245 and 5,294 for B18474. GPI anchored proteins were predicted with PredGPI using the general model and selecting proteins with high probability (> 99.90% specificity) (Pierleoni *et al.* 2008).

### Genome alignments

Shared synteny regions of at least 10 kb were identified using NUCmer v3.22 (Kurtz *et al.* 2004). Chromosomal rearrangements (translocations, inversions, and deletions) were identified from the alignment blocks based on alignment length, chromosome mapping, and orientation. Both long read and Illumina read alignments were manually inspected in IGV v2.3.72 (Robinson *et al.* 2011) to confirm that the rearrangement junctions are well supported in each assembly. Illumina sequences of 17 Clade II isolates (B13463, B11220, B11808, B11809, B14308, B12081, B12043, B12040, B12082, ERR2300774, ERR2842675, TWCC13846, TWCC13847, TWCC13878, TWCC50952, TWCC58191, and TWCC58362) (Lockhart *et al.* 2017; Chow *et al.* 2018, 2020; Sekizuka *et al.* 2019; Wasi *et al.* 2019) were aligned to the B8441 and B11245 genomes using BWA mem v0.7.12 (Li 2013) and deleted regions identified using CNVnator v0.3 (1 kb windows; *P*-value < 0.01) (Abyzov *et al.* 2011) and visually inspected using IGV v2.3.72 (Robinson *et al.* 2011).

### Variant identification

For variant identification and analysis, we included isolate sequences of Lockhart et al. (Lockhart *et al.* 2017) and five additional isolates from Clade II (B11808, B11809, B12043, B12081, and B14308) (Chow *et al.* 2018, 2020). We used FastQC and PRINSEQ (Schmieder and Edwards 2011) to assess the quality of reading data and perform read filtering low-quality sequences. Paired-end reads were aligned to the *C. auris* assembly strain B8441 [GenBank accession PEKT00000000.2; (Muñoz *et al.* 2018)] using BWA mem v0.7.12 (Li 2013). Variants were then identified using GATK v3.7 (McKenna *et al.* 2010) using the haploid mode and GATK tools *RealignerTargetCreator*, *IndelRealigner*, *HaplotypeCaller* for both SNPs and indels, CombineGVCFs, GenotypeGVCFs, GatherVCFs, SelectVariants, and Variant Filtration. Sites were filtered with Variant Filtration using “QD < 2.0 ‖ FS > 60.0 ‖ MQ < 40.0”. Genotypes were filtered if the minimum genotype quality < 50, percent alternate allele <0.8, or depth <10. Genomic variants were annotated and the functional effect was predicted using SnpEff v4.3T (Cingolani *et al.* 2012). The annotated VCF file was used to examine small polymorphisms (SNPs) and loss-of-function (LOF) mutations exclusively in isolates from Clade II adhesins.

## Data and resource availability

The whole-genome sequence and assemblies in this study are available in NCBI as follows: B11205 (CP060353-CP060359), B13916 (CP060374-CP060380), B11220 (CP043531-CP043537), B12043 (CP050666-CP050672), B11809 (CP050659-CP050665), B13463 (CP050652-CP050658), B12037 (CP060367-CP060373), B12631 (CP060360-CP060366), B17721 (CP060353-CP060359), B11245 (CP043442-CP043448), B12342 (CP060346-CP060352), and B18474 (CP050673-CP050679). Illumina sequence of Clade II isolates is available in BioProject PRJNA328792. Isolates are available from the CDC and FDA Antimicrobial Resistance (AR) Isolate Bank, https://www.cdc.gov/drugresistance/resistance-bank/index.html. Supplemental Material available at figshare: https://doi.org/10.25386/genetics.13759276.

## Results

### Chromosomal level features of *C. auris* clades

**Figure 1:**
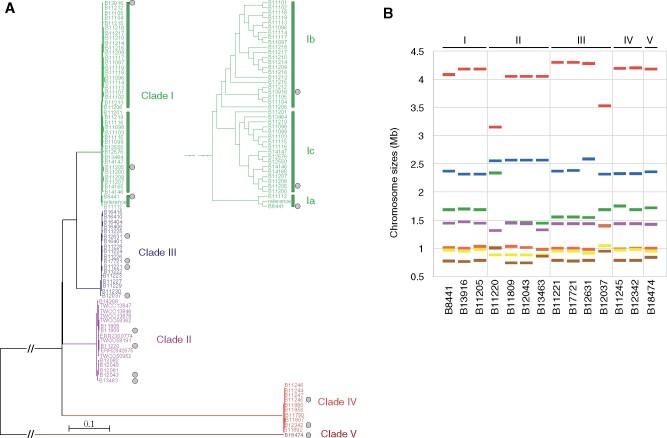
**Phylogenetic breadth and karyotypic variation in** *C. auris* **.** (A) Phylogenetic tree of 87 *C. auris* whole-genome sequences clustering into four major clades and the basal isolate B18474; potential fifth clade. Maximum likelihood phylogeny using 63,674 SNPs based on 1,000 bootstrap replicates. Isolate labels are color-coded by clade and gray circles indicated those isolates selected for chromosomal-level sequencing and assembly. (B) Chromosome sizes of fourteen isolates based on chromosome scale contigs.

To investigate genomic changes that could explain the emergence and different phenotypes observed between *C. auris* clades, we generated complete chromosome scale assemblies using long-read sequencing for twelve isolates from each of the four major clades—two Clade I, four Clade II, three Clade III, and two Clade IV—and for the only isolate reported to date from Clade V (B18474; NG19339). These isolates were selected to expand the phylogenetic breadth of genome assemblies for *C. auris* (Figure 1A). These include two isolates representing the two major causing outbreak lineages in Clade I, which are diverged from a small subclade that includes the commonly used B8441 reference genome; we denominated these as Clade la for the B8441 subclade and Clade lb and lc for the outbreak subclades, which each contain a specific drug-resistant mutation in lanosterol 14-alpha-demethylase (*ERG11*), with Clade Ib corresponding to *ERG11*^Y132F^ (isolate B13916) and Clade Ic corresponding to *ERG11*^K143R^ (isolate B11205). Four divergent Clade II antifungal susceptible isolates from different countries were sequenced (B11220 from Japan, B11809 from South Korea, B12043 from the United States, and B13463 from Canada). For Clades III and IV, five isolates were selected for sequencing to represent different countries and drug resistance profiles, including two from Clade IV (B11245 *ERG11*^Y132F^ from Venezuela and B12342 from Colombia) and three from Clade III (B17721 *ERG11*^F126L^ and B12631 *ERG11*^F126L; CNV^ from the United States and B12037 from Canada) (Table 1; Supplementary Table S1). These genomes were compared with the reference genomes of the isolates B8441 (Clade Ia) (Lockhart *et al.* 2017) and B11221 (Clade III; *ERG11*^F126L^) (Muñoz *et al.* 2018) (Figure 1A).

**Table 1:** Chromosomal-level assembly statistics

Isolate	Cladea	Country	Assembly size (Mb)	Chrs	Scaffolds	Genes	Telomeres	Accession
B8441	1a	Pakistan	12.37	7	15	5,419	NA	GCA_002759435.2
B13916	1b	UAE	12.4	7	7	5,305	12	CP060374-CP060380
B11205	1c	India	12.4	7	7	5,316	11	CP060353-CP060359
B11220	2	Japan	12.2	7	7	5,227	13	CP043531-CP043537
B12043	2	USA	12.2	7	7	5,242	13	CP050666-CP050672
B11809	2	South Korea	12.2	7	7	—	13	CP050659-CP050665
B13463	2	Canada	12.2	7	7	—	13	CP050652-CP050658
B11221	3	South Africa	12.7	7	20	5,520	NA	GCA_002775015.1
B12037	3	Canada	12.3	7	7	5,304	12	CP060367-CP060373
B12631	3	USA	12.5	7	7	—	13	CP060360-CP060366
B17721	3	USA	12.4	7	7	5,309	12	CP060353-CP060359
B11245	4	Venezuela	12.4	7	7	5,506	13	CP043442-CP043448
B12342	4	Colombia	12.4	7	7	—	13	CP060346-CP060352
B18474	5	Iran	12.4	7	7	5,294	12	CP050673-CP050679

a 1b = *ERG11* Y132F; 1c = *ERG11* K143R.

While seven chromosomes were assembled in each genome, substantial variation in chromosomes sizes was observed. While the three Clade I isolates contain similarly sized chromosomes, there was high variation across Clade II isolates, in particular B11220 (Figure 1B). Across the four Clade III assemblies, B12037 showed the most karyotypic variation. In the Clade III isolate B12631, a 220 kb increase in the size of chromosome 2 represents a segmental duplication and translocation of 267 kb of the region encompassing *ERG11* from chromosome 3 (Supplementary Figure S3). This variation in chromosome sizes in genome assemblies is consistent with a prior description of extensive karyotypic variation across *C. auris* isolates (Bravo Ruiz *et al.* 2019).

**Figure 2:**
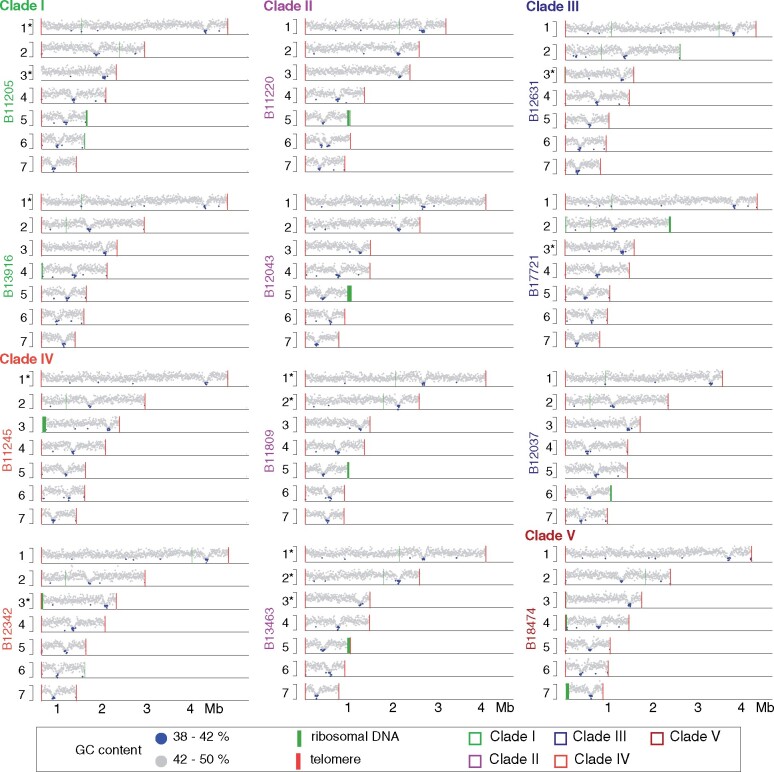
**Genome dynamics in** *C. auris* **.** Guanine-Cytosine (GC) content 0.38 to 0.50. GC content is color-coded <0.42 blue or >0.42 gray. Ribosomal DNA array are depicted as green lines and telomeres as red lines.

All genome assemblies consisted of 7 nuclear contigs corresponding to nearly complete chromosomes with telomeres at both ends. The one or two uncaptured contig ends correspond to ribosomal DNA in each assembly (Figure 2; Supplementary Table S1). For all genomes, each chromosome contains one discrete region for which guanine-cytosine (GC) content is markedly reduced (average GC content 41%; average length 85 kb; Figure 2; Supplementary Figure S1). These regions in each chromosome represent candidate positions of centromeres, as similar GC-poor troughs in *Pichia stipitis*, *Yarrowia lipolytica*, and the closely related species *Candida lusitaniae* had been shown to coincide with experimentally identified centromeres (Lynch *et al.* 2010; Kapoor *et al.* 2015). In only one case, two GC-poor troughs were detected; these are located in tandem in B11220 Chromosome 6 and appear to have resulted from a translocation associated with a large tandem duplication (145 kb) encompassing the candidate centromere (Figure 2; Supplementary Figure S2). As large ribosomal DNA arrays play an important role in genome dynamics, we examined each assembly for rDNA arrays and identified at least one large rDNA array located at a chromosome end, ranging in size from 25 kb to 85 kb (Figure 2). We found that the rDNA arrays appear dynamic in *C. auris* as their location varies between isolates, even those from the same clade. In the Clade I isolates B11205 and B13916, the rDNA array is located at the end of different chromosomes, 4 or 5, respectively. While the rDNA position appears conserved in Clade II isolates, located at the end of chromosome 5, the location varies in isolates from Clade III (B17721 and B12037, chromosome 2 and 6, respectively) and is found at a different location in Clade IV (B11245, chromosome 3) and Clade V (B18474, chromosome 7) (Figure 2). This variation suggests that recombination may be frequently linked to the rDNA array or possibly to chromosome ends.

### Large chromosomal rearrangements in *C. auris* Clade II

**Figure 3:**
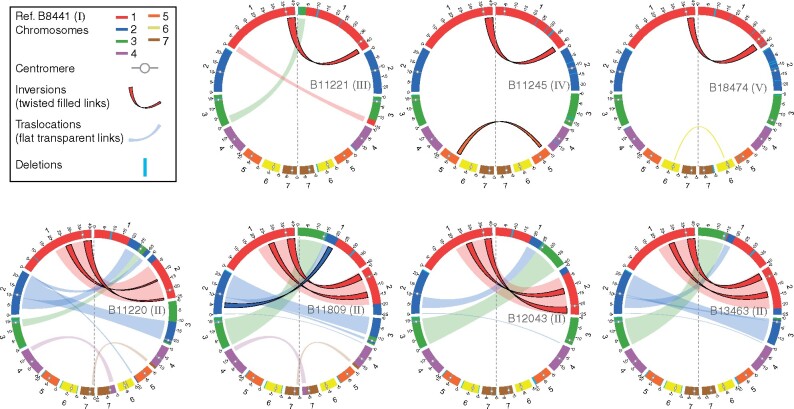
**Chromosomal rearrangements in** *C. auris* **.** Circos plots showing syntenic chromosomes by color and links for inversions (twisted filled links) and translocations (flat transparent links) using B8441 (Clade I) as reference compared to B11221 (Clade III), B11245 (Clade IV), and B18474 (Clade V) in the top row. The lower row of Circos plots depicts B8441 compared to four Clade II isolates (B11220, B11809, B12043, and B13463). Scaffolds/contigs to chromosome mapping for these genome assemblies is included in Supplementary Table S1.

While the number of chromosomes is conserved across isolates and the total genome size is similar (ranging from 12.2 to 12.5 Mb), chromosome lengths can differ substantially. This is most pronounced in Clade II isolates, for which chromosome sizes which can vary by up to 1.1 Mb relative to those from other clades, even the distantly related Clade V isolate (B18474) (Figure 1B; Supplementary Table S1). Based on whole-genome alignments, we found that differences in chromosomes lengths are largely due to large (>10 kb) interchromosomal rearrangements between isolates (Figure 3). Each Clade II isolate has undergone 2 to 3 inversions and 4 to 8 translocations compared to B8441 (Clade Ia), resulting in large changes in chromosome size. While these translocations are commonly found in the largest chromosomes 1, 2, and 3, the translocated segments differ between isolates in size and location, suggesting recent recombination (Figure 3). Chromosomal breakpoints in Clade II isolates in chromosomes 2, 3, 4, and 7 are adjacent to candidate positions of centromeres (Figure 3, Supplementary Figure S1). Inter-chromosomal recombination near centromeres, which has resulted in a substantially different karyotype in Clade II, has previously been observed in *Candida tropicalis* (Chatterjee *et al.* 2016).

In B11220, in addition to the translocation close to a tandem duplication of the candidate centromere, there was one rearrangement that resulted in an internal telomeric repeat array. This region is located on Chromosome 4, 22.7 kb from the chromosome end (Supplementary Figure S4). As telomeric repeats are otherwise only found at chromosome ends, the presence of this unusual structure in B11220 suggests an additional sign of increased genome instability. Comparison of B11220 with a nearly complete assembly of JCM 15448 (Sekizuka *et al.* 2019), a clonally related isolate with only 73 SNP differences, suggests a recent origin of the Chromosome 7 centromeric duplication, which is not present the JCM 15448 genome (Supplementary Figure S2). Inspection of read support for a potential fusion of B11220 contigs1 and 2 in JCM 15448 contig1 (BGOX01000001.1) suggests this is a mis-join in JCM 15448, not supported by read alignments (Supplementary Figure S5). These differences between closely related isolates in Clade II further support increased genome instability in this clade relative to Clades I, III, IV, and V.

### Outbreak-causing clades have highly syntenic stable genomes

While isolates from each *C. auris* clade associated with global outbreaks (I, III, and IV) have limited genetic diversity, we found evidence that intra-chromosomal rearrangements, particularly inversions, could occur between closely related isolates. By comparing Clade I isolates representing each of the subclades (Ia, Ib, and Ic), we identified three inversions. One inversion was found in chromosome 1 (scaffold01: 2720075-3096521) in B8441 (subclade 1a); the genes adjacent to the upstream breakpoint included B9J08_003891 (a small predicted protein-coding gene with similarity to the transcription factor *MRR1*) and B9J08_003892 (*ORC4*; a subunit of the origin recognition complex). The second breakpoint was located adjacent to the 3’ end of the transcriptional regulator *MRR1* (B9J08_004061), producing a truncated coding region relative to the full-length copy of *MRR1* in Clade III (CJI97_003963). An inversion in B11205 (subclade lc, *ERG11*^K143R^) is located in chromosome 1 (768159-904752) and the breakpoints are both adjacent to predicted proteins similar to the *C. albicans* Zorro3 retrotransposon gene *FGR14* (B9J08_003706 and B9J08_004248). Lastly, an inversion in B13916 (subclade l b, *ERG11*^Y132F^) is located in chromosome 5 (364491-767207); the breakpoints are most closely linked to the genes B9J08_004620 (*RNA15*)—B9J08_004798 (*ARG3*) and B9J08_004621 (*PTI1*)—B9J08_004799. These subclade-specific rearrangements, likely mediated by sequence similarity between the breakpoints, may result in differences in gene function, such as *MRR1* for subclade la, or possibly in gene regulation for other inversions.

Whole-genome alignments were also used to explore genome structure differences in Clade III. Most isolates from Clade III are azole-resistant and carry the *ERG11*^F126L^ (represented by B11221 from South Africa; B17721 and B12631 from the United States); genomes of these isolates appear highly syntenic. However, a divergent and drug susceptible isolate (B12037 from Canada, Figure 1A) showed higher karyotypic variation, largely due to four translocations (Supplementary Figure S6). In addition, this genome has undergone four large deletions at subtelomeric regions, at the beginning of chromosomes 6 and 7 and the end of chromosomes 4 and 5. In addition, we closely examined rearrangements associated with copy number variation in *ERG11*, one of the mechanisms contributing to azole-resistance in *C. auris* (Muñoz *et al.* 2018; Bhattacharya *et al.* 2019) predominantly observed in Clade III isolates (Chow *et al.* 2020). We found using both short read mapping and chromosomal assembly that B12631 has two copies of a ∼200kb region encompassing *ERG11*. Notably, one of the duplications resulted in a translocation of the *ERG11* region to the beginning of chromosome 2 (Supplementary Figure S3).

Two isolates from different countries in Clade IV were also examined. Comparison of chromosomal assemblies of the two Clade IV isolates, B11245 (Venezuela; *ERG11*^Y132F^) and B12342 (Colombia), revealed a large inversion in chromosome 1 (2481613–2925007). Both breakpoints are located in intergenic regions; the adjacent genes include B9J08_002936 (*RMS1*)—B9J08_002933 and the efflux transporter *BOR1* (B9J08_002715) and a predicted protein with similarity to retrotransposons (B9J08_002717).

These intraclade chromosomal changes, including in closely related isolates, suggests that the *C. auris* genome can rapidly undergo rearrangements. Some of these events appear to be associated with retroelements, suggesting nonallelic homologous recombination can occur between these small elements. While this appears to be a frequent mechanism of variation between isolates, isolates in this study were selected to maximize phylogenetic representation, and the wider frequency and impact on clinical phenotypes need to be established. In addition, these alterations in *C. auris* karyotype, most dramatically of Clade II, likely serve as a barrier to the production of viable progeny following mating and recombination.

### Large subtelomeric deletions and decay in *C. auris* Clade II

**Figure 4:**
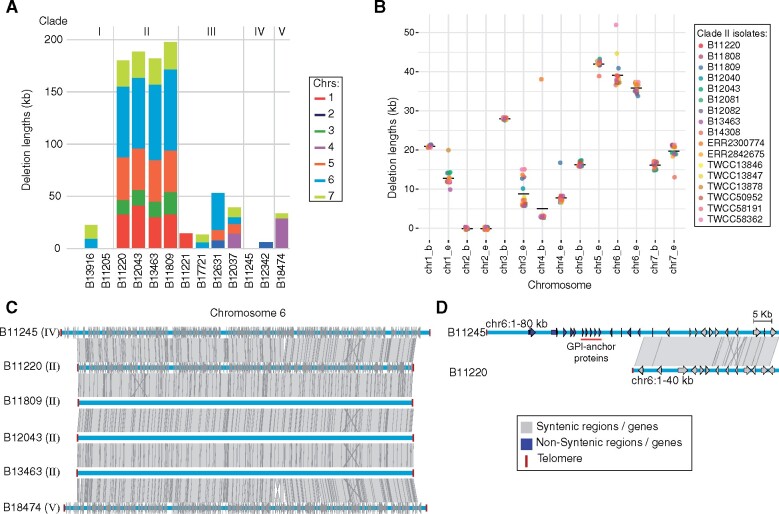
Subtelomeric decay in *C. auris* Clade II**.** (A) Length of the deleted sequences in subtelomeric regions in thirteen *C. auris* genomes. (B) Length of the deleted sequences in subtelomeric regions in seventeen isolates from Clade II. (C) Chromosome wide synteny between B11245 (Clade IV) and B11220 (Clade II). Chromosome 6 includes telomeres at both ends in both isolates (dark red square). Shared synteny regions based on genome alignment (blastn) are depicted in gray vertical blocks connecting the chromosome regions. Depicted genes in blue and light gray arrows showing the direction of transcription are color-coded according to the location in conserved (gray) or nonconserved (blue) regions. (D) Comparison corresponds to a zoom in of the subtelomeric region depleted in Clade II (B11220), which encompasses *auris*-clade specific adhesins in tandem (red line).

In addition to rearrangements, we identified large genomic regions that were missing in one or more clades, predominantly subtelomeric regions that are absent in Clade II isolates relative to Clades I, III, IV, and V. Comparing the Clade I (B8441) and Clade II (B11220) genomes, we identified 11 large regions (>5 kb) absent in Clade II that encompassed 226 kb and 74 genes; 10 of these 11 regions are subtelomeric in B8441 (Figure 4A; Supplementary Table S2). Comparing B8441 and B11220 with assemblies of Clade III (B11221) and Clade IV (B11245), we confirmed that these regions were also subtelomeric and only absent in Clade II isolates. These subtelomeric deletions are a common feature of isolates from Clade II, as these regions are also absent in the genome assemblies of other Clade II isolates from Canada, South Korea, and United States (Figure 4A). This is also apparent across 13 additional Clade II isolates from Japan (Sekizuka *et al.* 2019; Wasi *et al.* 2019), South Korea (Chow *et al.* 2020), Malaysia (ERR2300774), and the United States (Chow *et al.* 2018, 2020), based on alignment of Illumina sequence to the Clade I and IV references (Figure 4B). The length of the telomeric deletions varies by a few kilobases between Clade II isolates (Figure 4B). In addition, further decay might be impeded by the presence of homologs of genes shown to be essential in *C. albicans* at most Clade II chromosome ends (Supplementary Table S3). In addition, we observed differences in the length of subtelomeric regions even between samples from the same patient (Supplementary Figure S7). Three Clade II samples collected from a colonized patient over the course of one month (B12040, B12043, and B12082) (Chow *et al.* 2018) are highly clonal based on genomic analysis, with less than seven SNP differences between each pair. While these samples displayed subtelomeric deletions typical of Clade II, B12040 displayed a larger deletion at the end of chromosome 3; a region of 10 kb was deleted whereas only 6 kb was deleted in the two other clonal isolates from this same patient. The additional deleted region includes a putative alpha-1,3-mannosyltransferase (*MNN1*; B9J08_001516; Supplementary Figure S7). Mannosyltransferases play an important role in cell wall composition and immune recognition in *C. albicans* (Hall and Gow 2013) and *C. auris* (Bruno *et al.* 2020). This suggests that structural variation can contribute to intrahost variation of *C. auris* and may occur over a short period of time.

**Figure 5:**
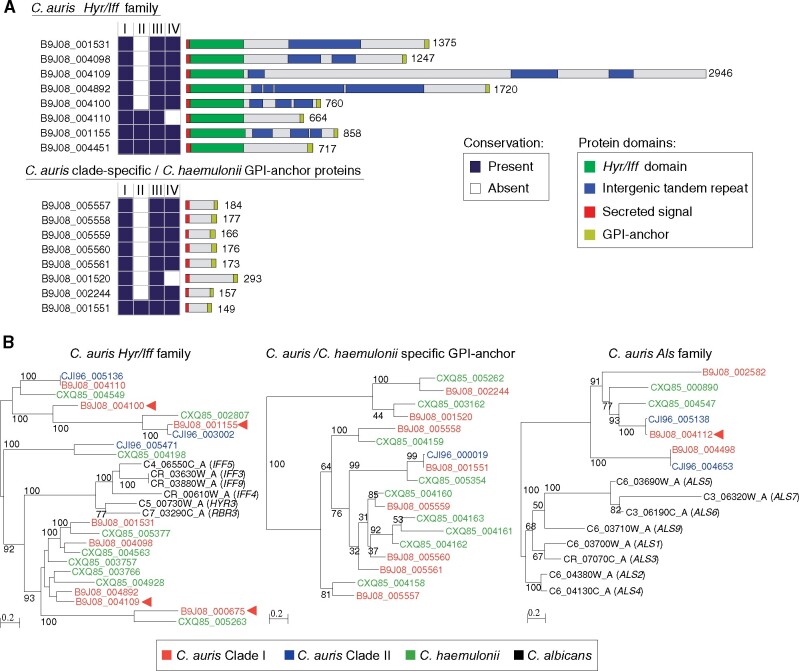
**Differences in the repertoire GPI-anchor proteins in** *C. auris* **.** (A) Conserved domains in two clusters of GPI-anchor found in *C. auris* B8441. (Top) *Hyr/Iff* GPI-anchor family and (bottom) *C. auris* clade-specific GPI-anchor protein. Conservation across *C. auris* isolates representing Clades I, II, III, and IV is color-coded indicating whether the gene is present (dark blue) or absent (white). (B) Phylogenetic analysis of the *Hyr/Iff* GPI-anchor family (left), the *C. auris* clade-specific GPI-anchor family (middle) and *Als* GPI-anchor family (right) using one representative isolate for *C. auris* Clades I and II, *C. haemulonii* and *C albicans*. Gene IDs are color-coded by species/isolate.

**Figure 6:**
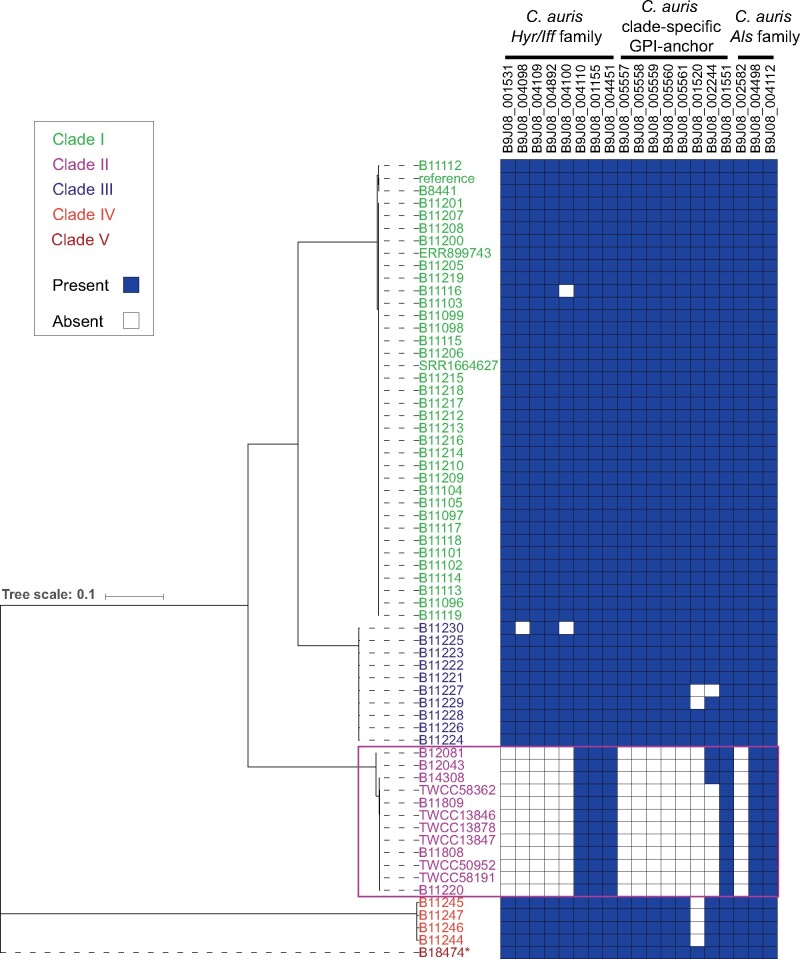
Conservation of GPI-anchor proteins across *C. auris* clades**.** Phylogenetic tree of 64 *C. auris* isolates from Clades I, II, III, IV, and V. Right panel depicts the conservation (present: blue; absent: white) of three families of adhesins. *B18474 a.k.a. NG19339.

To search for genetic variation that could explain these dramatic changes in genome integrity in Clade II, we identified mutations found exclusively in Clade II isolates (see Section Methods). These included 37 genes with loss-of-function mutations only found in Clade II, and only one of these impacted a gene involved in chromosome or DNA stability, a nonsense mutation near the start of *DCC1* (B9J08_000232) (amino acid change=Y10*; codon change=taC/taG; Supplementary Table S4). *DCC1* is a member of an alternate RFC complex involved in sister chromatid cohesion and telomere length maintenance (Askree *et al.* 2004). While the role of *DCC1* in *C. auris* has not been tested, gene deletion in *Saccharomyces cerevisiae DCC1* results in shorter telomeres (Askree *et al.* 2004) and genome instability (Yuen *et al.* 2007); this profile differs from that observed in hypermutator isolates with defects in DNA repair. This suggests that this naturally occurring loss of function mutation in *DCC1*, found in all Clade II isolates, might contribute to instability of heterochromatin, leading to shorter telomeres and genome rearrangements close to centromeres in this clade.

### Basally branching Clade V isolate is highly syntenic with outbreaks causing clades

The initial description of an isolate from Iran (B18474) corresponding to a fifth clade found that it appeared highly diverged from isolates from each of the other four clades (Chow *et al.* 2019). This drug-sensitive isolate came from a case of ear infection, properties shared with Clade II isolates (Chow *et al.* 2019). Using SNP-based analysis and whole-genome alignment of a *de novo* assembly, we confirmed that B18474 is distantly related to isolate from all other clades, yet the genome is highly syntenic with those of isolates from Clades I, III, and IV but not Clade II. Relative to B8441 (Clade I), B18474 (Clade V) only displayed two inversions (Figure 3) and one large deletion of 35 kb at the beginning of chromosome 4 (Figure 4A). This region is not deleted in any Clade II isolate examined, supporting an independent origin. This deleted region encompassed eleven genes, including three tandem copies of putative nicotinic acid transporters (*TNA1*), an acylglycerol lipase (*MGL2*) and the *SKS1* putative ser/thr kinase involved in glucose transport.

As B18474 appeared at a basally branching position compared to the other *C. auris* clades, we also examined the relationship of this isolate to those of species from the *Candida haemulonii* complex using a phylogenetic analysis of six genes (Supplementary Figure S8). In this phylogenetic tree, B18474 appears closely related to *C. auris* Clades I, II, III, and IV and highly divergent from the *C*. *haemulonii* species complex, supporting this isolate as a basally branching clade within *C. auris*. As hybrids have been reported for other *Candida* species, we measured the allele sharing between B18474 and all isolates from each of the other clades to evaluate if it could be a hybrid or potentially a mixed sample. The number of fixed alleles between B18474 and isolates from Clades I, II, III, and IV is 255,014, 263,799, 259,647, and 275,146, respectively. The roughly equal number of clade diagnostic alleles present in B18474 supports that it is similarly diverged from each of the four other clades; hybrid isolates would be expected to share a higher proportion of diagnostic alleles with parental lineages, as has been demonstrated in *Cryptococcus neoformans* for example (Rhodes *et al.* 2017). This is fivefold times higher than the number of fixed alleles between Clades I and II, which are the closest clades, and 1.5-fold times higher than the number of fixed alleles between Clades I and IV. In addition, B18474 has 176 K singleton SNPs, which is sevenfold times higher than the next isolate with the most unique SNPs. Although there is a single isolate from Clade V, the high number of private SNPs and similar number of fixed alleles shared with each of the four other clades supports that B18474 diverged before the separation of the four clades.

Based on read mapping and whole-genome alignment B18474 appeared heterothallic with mating type a similar to Clades I and IV isolates (Supplementary Figure S8). This isolate conserved the L-rhamnose cluster similar to Clade III isolates. Clade III isolates are able to assimilate L-rhamnose, but not Clades I, II, and IV isolates, which have deleted the L-rhamnose cluster (Ambaraghassi *et al.* 2019). The fact that this cluster is conserved in Clade V supports that in *C. auris* the absence of this cluster in Clades I, II, and IV is a loss rather than a gain in Clade III.

### Depleted *Hyr/Iff* and species-specific cell wall protein families in *C. auris* Clade II

The subtelomeric regions deleted in Clade II likely contribute to the phenotypic differences of this clade. Notably, these include the loss of fourteen candidate adhesins present in Clades I, III, IV, and V (Figure 5 and 6). These candidate adhesins are divided into two sets of genes that encode predicted GPI anchors and secretion signals, one set sharing sequence similarity to *C. albicans* adhesins from the *Hyr/Iff* family and a second set of tandemly arrayed genes only found in *C. auris* and the closely related species *C. haemulonii and C. duobushaemulonii* (Figure 5; Supplementary Table S5). The *Hyr/Iff* gene family was previously noted to be the most highly enriched family in pathogenic *Candida* species and has been associated with cell wall organization and virulence (Butler *et al.* 2009; de Groot *et al.* 2013). Six of eight *Hyr/Iff* proteins found in *C. auris* contain intergenic tandem repeats, which can vary in copy number and modulate adhesion and virulence (Bates *et al.* 2007; de Groot *et al.* 2013); five *Hyr/Iff* genes are deleted in Clade II isolates (Figure 5A; Supplementary Table S5). The second set of candidate adhesins are small proteins (149–293 amino acids) with serine/threonine-rich regions (9.0–17.3% Serine; 17.5–22.8% Threonine), and are tandemly located in subtelomeric regions conserved in Clades I, III, IV, and V, but absent in Clade II (Figure 2, 5A, and B; Supplementary Table S5). These proteins are conserved in species from the *C. haemulonii* complex but not more distantly related species (Figure 5B). The subtelomeric location and serine/threonine-rich region are properties shared with *C. glabrata EPA* adhesins (De Las Peñas *et al.* 2003), and the expansion of *EPA* adhesins has been linked to the emergence of the ability to infect humans in the *C. glabrata* lineage (Gabaldón *et al.* 2013). Several of the genes in deleted regions of Clade II [three of the *C. auris*-clade specific adhesins, one *HYR-like* gene, and other cell wall associated proteins (*ALS4*, *CSA1*, and *RBR3*)] were transcriptionally up-regulated in developing *C. auris* biofilms (Kean *et al.* 2018; Supplementary Table S2), suggesting they play a role in biofilm formation. Other changes associated with cell wall and mannose modifications include the loss of another putative alpha-1,3-mannosyltransferase (*MNT4*; B9J08_004891) and a loss of function mutation in all Clade II isolates in the N-acetylglucosamine (GlcNAc) kinase (*HXK1*; B9J08_003836; amino acid change=Q101*; codon change=Caa/Taa; Supplementary Table S4). The loss of adhesin-like genes, as well as genes involved in cell wall modifications in Clade II isolates, could help explain epidemiological differences between this clade and the three other clades that are more commonly observed.

### Clade-specific cell wall proteins and drug-related genes have signatures of selection

We examined the genomes of *C. auris* for signatures of selection using the composite likelihood ratio (CLR) test (Nielsen *et al.* 2005) and scanned genes for signatures of selective sweeps in *C. auris* Clades I, III, and IV. We did not carry out this analysis in Clades II and V due to the limited number of isolates from these clades. Overall, the genes in the top 5% of CLR values in Clades I, III, and IV fall into two categories. One group of genes associated with drug resistance, including the drug targets *ERG11* and *FKS1*, the transcriptional regulator *TAC1b* and the drug efflux pump *CDR1* (Table 2). The second group of genes related to invasion and biofilm formation, including the cell wall proteins *RBR3*, *HYR3*, *IFF6*, and *ALS4* (Figure 5), the secreted yeast wall protein *YWP1* and the transcriptional regulators *WOR2*, *ZCF32*, and *OPI1* (Table 2). Notably, some of these genes display signatures of selection in all three clades or in two of the three (Table 2), suggesting shared and clade-specific mechanism of host and drug adaptation. The drug targets *ERG11* and *FKS1* and the cell wall proteins *HYR3* (B9J08_000675) and *RBR3* (B9J08_004100) displayed signatures of selection in all three clades. Drug resistance mutations in *ERG11* (Lockhart *et al.* 2017; Chowdhary *et al.* 2018 p. 350; Chow *et al.* 2020) and *FKS1* (Chowdhary *et al.* 2018; Chow *et al.* 2020) have been reported in Clades I, III, and IV. In addition, *TAC1b* and *CDR1* appear under selection in Clades I and IV, but not Clade III, suggesting a stronger contribution of variants in these genes to azole-resistance in these clades. This is supported by recent work that demonstrated that mutations in *TAC1b* contribute to fluconazole resistance (Rybak *et al.* 2020) and that expression of *CDR1* is differentially regulated between isolates from Clades III and IV (Mayr *et al.* 2020). Together, this suggests shared and clade-specific evolution of both drug-resistance and cell wall proteins that may impact host interaction. These candidate cell wall proteins are strong candidates for experimental validation to better understand the high rate of transmission of the *C. auris* outbreak clades within health care facilities and the ability to persist in colonized patients and on plastic surfaces.

**Table 2.** Genes in the top 5% of composite likelihood ratio (CLR) test values of selection

Clade	Gene ID	Category	Gene	Description
Clades I, III, and IV	B9J08_001448	Drug target	*ERG11*	Lanosterol 14-alpha-demethylase
	B9J08_000964	Drug target	*FKS1*	Beta-1,3-glucan synthase
	B9J08_000675	Cell wall	*RBR3*	Cell wall adhesin-like protein
	B9J08_004100	Cell wall	*HYR3*	Putative GPI-anchored adhesin-like protein
Clades I and III	B9J08_000336	Cell wall	—	Mucin-like protein
	B9J08_004109	Cell wall	*RBR3*	Cell wall adhesin-like protein
	B9J08_000045	Chromatin remodeling	*ISW2*	Translocase involved in chromatin remodeling
	B9J08_000092	Intracellular transport	*DYN1*	Dynein; microtubule motor protein
	B9J08_004769	Protein degradation	*UBI4*	Ubiquitin
	B9J08_001715	Phosphorylation	*URK1*	Uridine/cytidine kinase
	B9J08_004988	Transcription factor	—	Predicted transcription factor
	B9J08_005188	Transcription factor	*TIF4631*	Putative translation initiation factor eIF4G
	B9J08_001729	Transcription factor	*ZCF32*	Zn(II)2Cys6 transcription factor (biofilm)
	B9J08_002715	Transporter	*BOR1*	Borate efflux transmembrane transporter
	B9J08_002864	Transporter	*NGT1*	N-acetylglucosamine (GlcNAc)-specific transporter
	B9J08_003323	Transporter	*YOR1*	Plasma membrane ABC transporter
Clades I and IV	B9J08_000164	Transporter	*CDR1*	Multidrug transporter of ABC superfamily
	B9J08_004718	Transporter	*OSH2*	OxySterol binding protein Homolog
	B9J08_004820	Transcription factor	*TAC1b*	Zn(2)-Cys(6) transcriptional activator (efflux pumps)
	B9J08_002136	Transcription factor	*WOR2*	Zn(II)2Cys6 transcription factor (white-opaque switching)
Clades III and IV	B9J08_003550	Cell wall	*YWP1*	Secreted yeast wall protein
	B9J08_001155	Cell wall	*IFF6*	Putative GPI-anchored adhesin-like protein
	B9J08_004112	Cell wall	*ALS4*	Putative GPI-anchored adhesin-like protein
	B9J08_001181	Transcription factor	*OPI1*	Leucine zipper transcriptional regulator

## Discussion

In this study, we generated and analyzed 12 complete, telomere-to-telomere, *C. auris* genomes representing the phylogenetic breadth of the five phylogenetic clades. By comparing these genomes, we characterized a major genetic difference between isolates from Clade II compared to those from Clades I, III, IV, and V. We discovered that the genomes of Clade II isolates, which are often drug susceptible and associated with ear infections, have increased chromosomal rearrangements, genome instability, and telomere shortening. We found that *C. auris* subtelomeric regions are enriched in cell wall proteins, which are largely lost in Clade II isolates. These cell wall proteins fall into three families that are also targets of selection in Clades I, III, and IV, along with other predicted cell wall proteins and genes involved in drug resistance. The loss of these cell wall proteins in Clade II potentially impacts adhesion and biofilm formation, and may help explain the lower virulence and potential for transmission in Clade II isolates. While the isolates from the outbreak-causing Clades I, III, and IV have very low intra-clade genetic diversity, comparison of complete genomes identified large genomic changes that will be important to further evaluate for an impact on host interaction and drug resistance. This analysis underscores the importance of generating genome assemblies of diverse isolates to understand the impact of genomic plasticity across a population.

A major challenge in managing *C. auris* infections is that unlike other *Candida* this species can be easily transmitted within health care facilities. This may be accelerated by the ability to persist on plastic surfaces common in health care settings (Welsh *et al.* 2017). In addition, the ability of *C. auris* to form biofilms might enhance the capacity of *C. auris* to colonize patients’ skin for long periods of time and survive in healthcare setting even after cleaning, further increasing transmission and potentiating outbreaks. These are common features of isolates from Clades I, III, and IV that are the primary cause of invasive infections and have each caused outbreaks in healthcare settings (Welsh *et al.* 2019). The identification of candidate cell wall proteins and mannosyltransferases that are absent in the highly rearranged genome of Clade II, highlights the major differences that can occur between otherwise closely related isolates of a species. Understanding how *C. auris* survives in healthcare settings and on patients’ skin, and how different clades respond to different stressors, is essential to understand its pathobiology, however, studies to date have only tested small numbers of samples for each of the four major clades. Overall, *C. auris* isolates showed salt tolerance and thermotolerance up to 42°C, with a subset showing an aggregating phenotype (Borman *et al.* 2016; Sherry *et al.* 2017). A recent study of sixteen *C. auris* isolates found they were generally resistant to the cationic, oxidative, nitrosative and cell wall stresses, however, the one Clade II isolate tested was highly susceptible to Calcofluor White and Congo red (Heaney *et al.* 2020), suggesting that Clade II isolates are more susceptible to cell wall stress. A pair of recent studies showed that Clade II isolates from Japan and South Korea were more sensitive to UV-C killing than Clades I, III and IV isolates originating from Venezuela, Spain and India (de Groot *et al.* 2019; Chatterjee *et al.* 2020). Although most Clade II isolates are drug susceptible isolated from ear infection, some isolates appear to cause fungemia and acquire azole resistance (Lee *et al.* 2011), suggesting that, even with a highly rearranged genome and large subtelomeric losses, this clade has the potential to cause severe, drug-resistant infections.

Our study highlights the importance of more widely evaluating phenotypic variation across *C. auris* clades. It will be important to understand how genomic variation including clade-specific differences may affect properties such as biofilm formation, adhesion, invasion and damage to elucidate the basis of infection, colonization, and transmission of *C. auris*. Future studies are also needed to test the direct role of subtelomeric gene families of potential adhesins in human colonization and virulence. Recent studies have found that some predicted cell wall protein genes are differentially regulated under physiologically-relevant conditions, including the candidate adhesins in subtelomeric regions deleted in Clade II (Kean *et al.* 2018; Yue *et al.* 2018). One adhesin homolog of *C. albicans ALS4* (B9J08_004112) is differentially expressed during *C. auris* filamentous growth (Yue *et al.* 2018), and other GPI-anchored cell wall genes (*IFF4*, *PGA26*, *PGA52*, and *HYR3*) and potential ALS adhesin (B9J08_002582) were upregulated during *C. auris in vitro* biofilm formation compared to planktonic cells (Kean *et al.* 2018). In addition, two *ALS* family orthologs, were expressed in biofilms (Kean *et al.* 2018). In *C. albicans*, *ALS3* was used to generate a vaccine against *C. albicans* that cross-protects against *C. auris* (Singh *et al.* 2019); *ALS3* (B9J08_004498) is conserved in all clades, and is even found in Clade II and the only isolate described to date from potential Clade V, suggesting this vaccine may be broadly effective.

The single isolate from Iran that represents a potential fifth clade was hypothesized to be related to Clade II, based on shared drug susceptibility and causing ear infections. We confirmed B18474 is a basally branching isolate relative to the four major clades and is not closely related to the *C. haemulonii* species complex. As the genome was found to be highly syntenic with those of isolates of Clades I, III, and IV, this supports that the high rate of genome rearrangements and subtelomeric loss are unique features of Clade II isolates. Once more isolates are reported from this potential fifth clade, it would be possible to confirm if the ear infection and drug susceptibility are general patterns of this clade; isolates could also be sequenced to measure genetic diversity and allele sharing for Clade V compared to the other clades.

These chromosomal assemblies have highlighted the genomic instability and gene loss that has occurred in Clade II isolates as well as how structural variation contributes to diversity in other clades. The lost cell wall proteins identified in our study are not well studied to date, even the *Iff/Hyr* family shown to be fast evolving in other *Candida* species. These cell wall proteins could have implications for the observed differences in susceptibility to disinfectants, ability to persist outside human host, and transmission dynamics. Future experimental testing of these genes may help explain differences in the clinically important properties of these clades, including the severity of infection and propensity to cause outbreaks, which will help develop strategies to control this emerging fungal threat.

## Disclaimer

The use of product names in this manuscript does not imply their endorsement by the US Department of Health and Human Services. The finding and conclusions in this article are those of the authors and do not necessarily represent the views of the Centers for Disease Control and Prevention.
